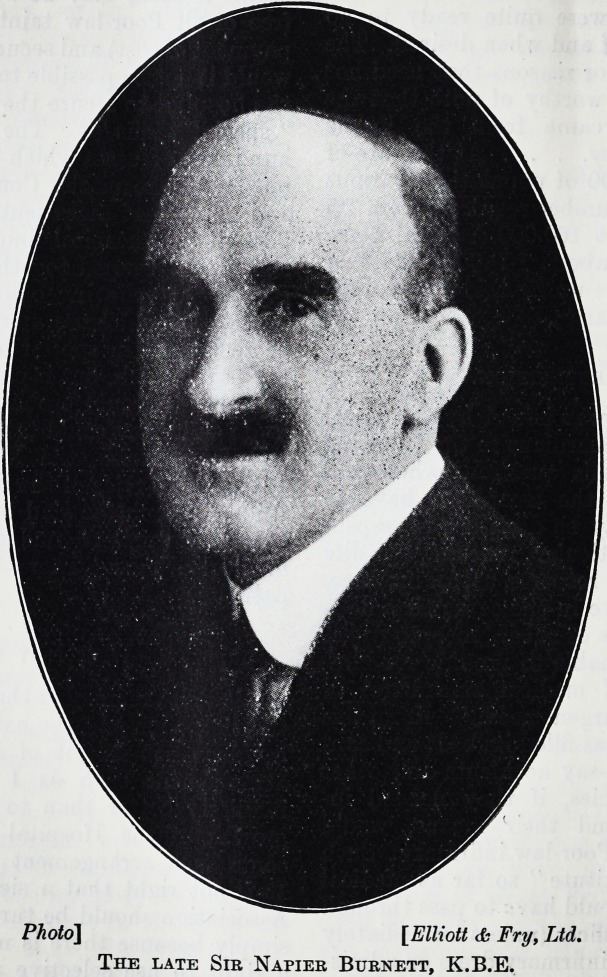# Sir Napier Burnett: An Appreciation

**Published:** 1924-02

**Authors:** Sydney Walton


### SIR NAPIER BURNETT: AN APPRECIATION.

By SYDNEY WALTON,

IN the January number of the HOSPITAL AND HEALTH REVIEW I chanced upon a sentence which might serve as the theme of the evangel proclaimed by the late Sir Napier Burnett in all his public speeches during recent years. "The medicine of the future will be mainly preventive medicine." He was the apostle of that truth, and with his own native gifts of exposition he did more than most men to set it chiming within the nation's thought. I sometimes wish he could have been set free from the bonds of desk and detail work in order to tour the country and set hearts aflame with the gospel and vision which thrilled in him like a passion. But apostleship was only part of his great qualities. He was a wise counsellor and a skilled organiser. In committee or conference men instinctively turned to Sir Napier for guidance. His calm, clear insight and swift, unerring diplomacy cut a path through many a perplexing problem and took us beyond the mists into the sunshine. In power to organise he had few peers, for he knew the deeper human secrets whence inspiration and gladness in our work derive. His sincerity and enthusiasm captured the minds and stirred the hearts of those with whom he came into touch.

*Photo*] [*Elliott & Fry, Ltd.*

THE LATE SIR NAPIER BURNETT, K.B.E.

### HIS LAST REPORT.

It was on Christmas morning that he fell on sleep after an illness lasting many months and bravely borne. He was only fifty-one years old, at the zenith of his hopes and in his golden prime. In the early summer of last year he completed his annual hospitals report which had almost become a classic. As I turn over its pages now, a pathos and sadness cling to them, for I now remember that the shadow of death was upon the author as he wrote. He would not give himself rest and holiday until that work was finished. He alone knew how ill he was as he wrestled with the statistics and analyses which make the Report so illuminating and so rich a document. When the volume was ready for the press he went abroad for a cure and returned to his native Aberdeenshire only to find that disease pursued him. He was many weeks in a nursing home in Edinburgh, and his wonderful heroism and sheer will to live promised at times a victory over the physical foes which beset him, but hope only flattered to deceive.

### WORK FOR THE BRITISH RED CROSS.

Those fifty years were crowded with noble thoughts and activities, especially the last ten of them—a decade of rare achievement for hospitals in war and peace. Called from his growing practice on Tyneside to the War Office, he served the State in his own skilled and modest way and with tireless energy. One day, in the course of his official duties, he went to St. Thomas's Hospital to pursue some inquiries. There he met Sir Arthur Stanley, to whom he told his dream about hospitals. It was a historic hour, for out of that talk there ultimately came the invitation to Sir Napier Burnett to be the Chief Executive Officer of the British Red Cross and the Order of St. John of Jerusalem. At heavy personal and financial sacrifice Sir Napier left Newcastle and gave his whole time and service to humane ministries.

### THE NEW CITY OF HEALTH.

He is gone, but his inspiration lives, and he leaves to us the heritage of a task which in humanity's name must be fulfilled. He saw in a vision the new city wherein health has overthrown the dark dominion of disease, and he did not cease from mental strife, nor did his sword sleep in its sheath, until he had spread among us the fire of his own dream and desire. Our greatest tribute to him should be an increasing devotion to the causes he loved and served.

## Figures and Tables

**Figure f1:**